# Multi-omics-driven biomarker discovery in autoimmune diseases: a comprehensive review

**DOI:** 10.3389/fimmu.2025.1652211

**Published:** 2025-12-08

**Authors:** Yi Zhang, Haofeng Xu, Lijuan Xu, Shasha Jiang, Yan Yu, Heping Zhao

**Affiliations:** Department of Clinical Laboratory, Honghui Hospital, Xi’an Jiaotong University, Xi’an, China

**Keywords:** autoimmune diseases, biomarker, multi -omics, machine learning, network analysis

## Abstract

Autoimmune diseases (ADs) exhibit complex heterogeneity and dynamic pathological mechanisms. Traditional biomarkers face numerous challenges in the diagnosis and treatment of ADs. However, the rapid development of multi-omics technologies and bioinformatics has not only deepened the understanding of the pathogenesis of ADs but also identified many novel diagnostic and therapeutic biomarkers with good diagnostic performance. These biomarkers are now beginning to overcome these limitations. This review systematically explores the discovery of novel biomarkers driven by multi-omics technologies such as genomics, epigenomics, transcriptomics, proteomics, metabolomics, and microbiomics, in response to the limitations of traditional biomarkers. It emphasises the significant importance of discovering novel biomarkers through multi-omics in the diagnosis and treatment of ADs, and proposes a concept from omics analysis to solving clinical problems, providing new directions for the diagnosis and treatment of ADs.

## 1 Introduction

Autoimmune diseases (ADs) are a heterogeneous group of disorders in which the immune system loses self-tolerance and mistakenly attacks host tissues. This loss of tolerance drives aberrant expansion and activation of autoreactive T and B cells, causing collateral damage to healthy organs (1). The prevalence of most ADs in the general population ranges from 0.1% to 1.0%, but for individuals with a first-degree relative affected by such a condition, the risk is five times higher (2). Even today, the diagnosis of ADs often remains a lengthy process, and the prognostic tools currently available can at times be limited (3). ADs are uniquely complex, posing formidable challenges to accurate prediction. Consequently, there has been a persistent demand for sensitive and specific markers that can signal therapeutic response, and permit real-time tracking of disease flare or remission. Biomarkers—molecules whose presence or abundance correlates with disease severity or other pathophysiological states—remain the central pillar of the field (4).

Conventional biomarkers still serve as the mainstay of diagnosis for many ADs (5, 6). Nevertheless, their clinical utility is constrained by modest sensitivity and specificity and by uncomfortably high false-positive rates—limitations that are reviewed in detail below (7, 8). Over the past two decades, however, multi-omics technologies and bioinformatics have advanced markedly. The discoveries of new biomarkers driven by multi-omics are now beginning to overcome these limitations. In systemic lupus erythematosus (SLE), for example, genomics, epigenomics, transcriptomics, proteomics, metabolomics and microbiome have collectively identified a host of novel biomarkers with superior diagnostic performance (9–13), several of which have already been validated (14, 15), thereby providing new directions for both diagnosis and therapy. By interrogating how genetic, molecular or microbial variation relates to distinct ADs, we have not only deepened our understanding of disease mechanisms but also reveal pathways that may harbour potential drug targets. Moreover, the recent expansion of multi-omics technologies is accelerating the discovery of novel diagnostic and therapeutic markers and, in some settings, enabling precise clinical stratification (16).

Addressing the limitations of traditional biomarkers in ADs, we have conducted a comprehensive review of the literature on the discovery of novel biomarkers driven by multi-omics. Particular emphasis is placed on biomarkers validated by methods such as machine learning, with a significant number of these biomarkers accompanied by performance metrics like the area under the curve (AUC). In the review, we analyse the importance of multi-omics in facilitating the discovery of novel diagnostic and therapeutic biomarkers for ADs and propose a concept for addressing clinical issues through omics analysis.

## 2 Traditional biomarkers in ADs: limitations and challenges

Owing to their complex pathogenesis and variable clinical phenotype, ADs remain difficult to manage (17). At present, traditional biomarkers are hampered by insufficient sensitivity and specificity, poor dynamic monitoring capacity, reliance on invasive procedures, vulnerability to non-disease-related confounders and overly restrictive diagnostic criteria (**Table 1**). The combination of multiple biomarkers can enhance diagnostic efficacy. The establishment of a multi-dimensional biomarker system—integrating genomics, epigenomics, proteomics, and metabolomics—represents a pivotal strategy to transcend the limitations of conventional single-biomarker approaches. Future efforts should focus on optimising biomarker combinations via machine learning algorithms and the development of non-invasive dynamic monitoring technologies to refine diagnostic and therapeutic precision in ADs.

**Table 1:** The diagnostic and therapeutic limitations of traditional biomarkers in ADs.

Disease	Traditional biomarkers	Limitations
Systemic Lupus Erythematosus (SLE) (5, 18, 19)	Anti-dsDNA, Anti-Smith, ANA, Complement C3/C4	1. Low sensitivity (Anti-dsDNA 33.3%, Anti-Smith 11.4%) 2. Insufficient specificity (ANA prone to false positives) 3. Unable to effectively identify seronegative patients 4. Weak association with disease activity 5. Unable to dynamically monitor disease progression
Lupus Nephritis (LN) (20–22)	Anti-dsDNA, Complement C3/C4, Urine Protein Quantification, Renal Biopsy	1. Low sensitivity (Anti-dsDNA AUC = 0.580) 2. Insufficient dynamic monitoring capability (Urine Protein Quantification is cumbersome) 3. Invasive (Renal Biopsy) 4. Interference from non-disease factors (Urine Protein false positives)
Paediatric Multiple Sclerosis (pMS) (23)	Oligoclonal Bands (OCBs), MRI	1. Low sensitivity (OCB only 50% in children) 2. Limited specificity (OCB prone to false positives) 3. Invasive (CSF detection requires lumbar puncture) 4. Insufficient dynamic monitoring capability 5. Interference from non-disease factors (MRI imaging affected by physiological changes)
Rheumatoid Arthritis (RA) (7, 8, 24)	Anti-CCP, RF, CRP, ESR	1. Low sensitivity (Anti-CCP 78%) 2. Insufficient dynamic monitoring capability (unable to reflect joint inflammation in real-time and identify early immune changes) 3. Insufficient specificity (RF 81.2%) 4. Lack of early diagnostic biomarkers 5. Limitations of single biomarkers
Anti-Neutrophil Cytoplasmic Antibody-Associated Vasculitis (AAV) (6)	ANCA, CRP, ESR	1. Low sensitivity (ANCA-negative patients account for 10-20%) 2. Insufficient dynamic monitoring capability (ANCA titre weakly associated with activity) 3. Limitations of classification criteria (reliance on single organ involvement) 4. Interference from non-disease factors (medications, infections)
Juvenile Idiopathic Arthritis-Associated Uveitis (JIAU) (25, 26)	ANA, Age, ILAR Subtype	1. Low sensitivity (ANA OR = 2.79) 2. Insufficient dynamic monitoring capability 3. Limitations of diagnostic criteria (heterogeneity in screening guidelines) 4. Interference from non-disease factors (gender, genetic heterogeneity) 5. Lack of individualised predictive capability
Multiple Sclerosis (MS) (23, 27)	Oligoclonal Bands (OCBs)	1. Low sensitivity (OCBs 83.8%) 2. Defects in detection methods (subjective, time-consuming) 3. Singularity of diagnostic criteria (unable to distinguish MS from other inflammatory diseases) 4. Disadvantage compared to novel biomarkers (kFLC parameters have higher AUC)

Insufficient sensitivity and specificity. Traditional biomarkers demonstrate marked deficiencies in sensitivity and specificity, contributing to unavoidable risks of misdiagnosis and diagnostic omission. Although anti-cyclic citrullinated peptide antibodies (anti-CCP) and rheumatoid factor (RF) exhibit high specificity for rheumatoid arthritis (RA), their sensitivity remains suboptimal, particularly in early-stage RA. Furthermore, RF may cross-react with other inflammatory conditions (7, 8). In lupus nephritis (LN), anti-dsDNA antibodies and complement C3/C4 levels are key indicators. However, approximately 20–30% of patients lack characteristic antibody titre fluctuations, complicating active lesion surveillance (20, 21). Methodological disparities in antineutrophil cytoplasmic antibody (ANCA) detection (indirect immunofluorescence versus ELISA) and seronegativity in some patients delay the diagnosis of ANCA-associated vasculitis (AAV) (6). Similarly, anti-nuclear antibodies (ANA), employed as a screening tool for juvenile idiopathic arthritis-associated uveitis (JIAU), exhibit poor positive predictive value and cannot discriminate between active and inactive disease states (25, 26). These biomarkers—whether utilised individually or combinatorially—fail to account for disease heterogeneity (e.g., subtype variations and seronegative phenotypes), underscoring the imperative for novel molecular biomarkers or multi-omics integration to improve diagnostic fidelity.
Limited capacity for dynamic monitoring. Traditional biomarkers exhibit significant constraints in dynamically tracking disease progression and therapeutic responses in clinical practice. For instance, in SLE, patients with negative conventional biomarkers and normal complement levels may still harbour undetected disease activity (18, 19), underscoring the inadequacy of existing indicators in identifying occult pathological changes. In LN assessment, although 24-hour urine protein quantification remains the gold standard, its procedural complexity and insensitivity to weakly positive results (e.g., 1+ or 2+ proteinuria) compromise early diagnostic efficacy (22). Similarly, JIAU monitoring systems fail to comprehensively delineate the dynamic evolution of disease risk, potentially masking subclinical inflammatory activity. For the inflammatory arthritis phase in the preclinical stage of RA, classical biomarkers such as erythrocyte sedimentation rate (ESR) and C-reactive protein (CRP) cannot elucidate lymphocyte subset imbalances or early immune microenvironment alterations (24), leaving clinical decisions devoid of precise immunological context. Collectively, these limitations highlight systemic deficiencies in current biomarker frameworks: an inability to integrate multi-dimensional biological data in real time, particularly in diseases governed by complex immunopathological mechanisms. Traditional unidimensional metrics are thus increasingly misaligned with the demands of precision medicine.
The impact of diagnostic criteria and procedures. Contemporary clinical diagnostic frameworks exhibit substantial limitations in the management of complex diseases, primarily characterised by significant heterogeneity in classification systems, cumbersome operational protocols, and the inherent constraints of invasive testing modalities that impede dynamic disease monitoring. SLE diagnosis, reliant on clinical criteria (e.g., Systemic Lupus International Collaborating Clinics or American College of Rheumatology classifications), may misclassify atypical presentations (5). The gold-standard renal biopsy for LN, while diagnostically indispensable, suffers from intrinsic invasiveness that precludes serial assessment and real-time tracking of disease progression (20). Similarly, in multiple sclerosis (MS), the detection of oligoclonal bands (OCBs) remains constrained by both subjective interpretive variability and protracted analytical processes, collectively compromising diagnostic efficiency (23, 27). Further compounding these challenges, AAV diagnosis is hampered by persistent discrepancies in guideline recommendations regarding optimal screening frequencies and age thresholds, resulting in diminished diagnostic consistency across clinical studies (6). These operational limitations not only affect the precision of clinical decision-making but also pose obstacles to the dynamic assessment of disease progression.
Inability to accurately reflect disease mechanisms. Conventional biomarkers exhibit fundamental limitations in elucidating the comprehensive pathophysiological cascades underlying complex disorders. This mechanistic shortcoming is particularly evident in JIAU, where current diagnostic parameters fail to incorporate critical immune cell population dynamics that directly correlate with disease evolution (26). Similarly, traditional biomarkers for pre-clinical phase of RA fail to account for lymphocyte alterations, thereby creating critical knowledge gaps in our understanding of disease progression pathways (24). Such systemic failures in capturing the immunological choreography of disease mechanisms not only obscure the fundamental nature of these conditions but also render therapeutic interventions imprecise, as clinical strategies cannot be optimally aligned with core pathological drivers.
Interference from non-disease factors. The analytical reliability of conventional biomarkers remains fundamentally constrained by confounding variables unrelated to underlying pathology, systematically distorting diagnostic assessments and impeding longitudinal disease monitoring. In LN, urinary protein quantification demonstrates susceptibility to transient perturbations induced by intercurrent infections or vigorous physical exertion, whilst renal functional parameters—most notably estimated glomerular filtration rate (eGFR)—exhibit inherent physiological dependency on age-related changes and somatic muscle mass, thereby obscuring the correlation between biomarker levels and actual histopathological progression (20). This vulnerability extends to neuroinflammatory disorders, where antecedent vaccinations or systemic infections alter cerebrospinal fluid (CSF) and serum profiles of inflammatory mediators, including CRP and leukocyte subpopulations, critically undermining diagnostic specificity in paediatric MS (pMS) (23). Furthermore, inter-individual biological variability across demographic parameters (age, sex) and comorbid states (obesity, subclinical infections) induces non-pathological fluctuations in established biomarkers such as CRP and RF, elevating risks of both false-positive and false-negative interpretations. Notably, therapeutic interventions themselves may introduce diagnostic artefacts, as evidenced by the capacity of non-steroidal anti-inflammatory drugs and corticosteroids to pharmacologically attenuate inflammatory cascades in RA, effectively dissociating biomarker profiles from genuine disease activity (7).

These persisting limitations in diagnostic sensitivity, specificity, and prognostic utility underscore the imperative for integrative diagnostic frameworks that synergise emerging biomarker modalities—spanning multi-omics analysis—with multidimensional clinical datasets, to achieve enhanced therapeutic stratification and personalised disease management.

## 3 Omics-driven discovery of novel biomarkers to promote the diagnosis and treatment of ADs

**Figure 1:**
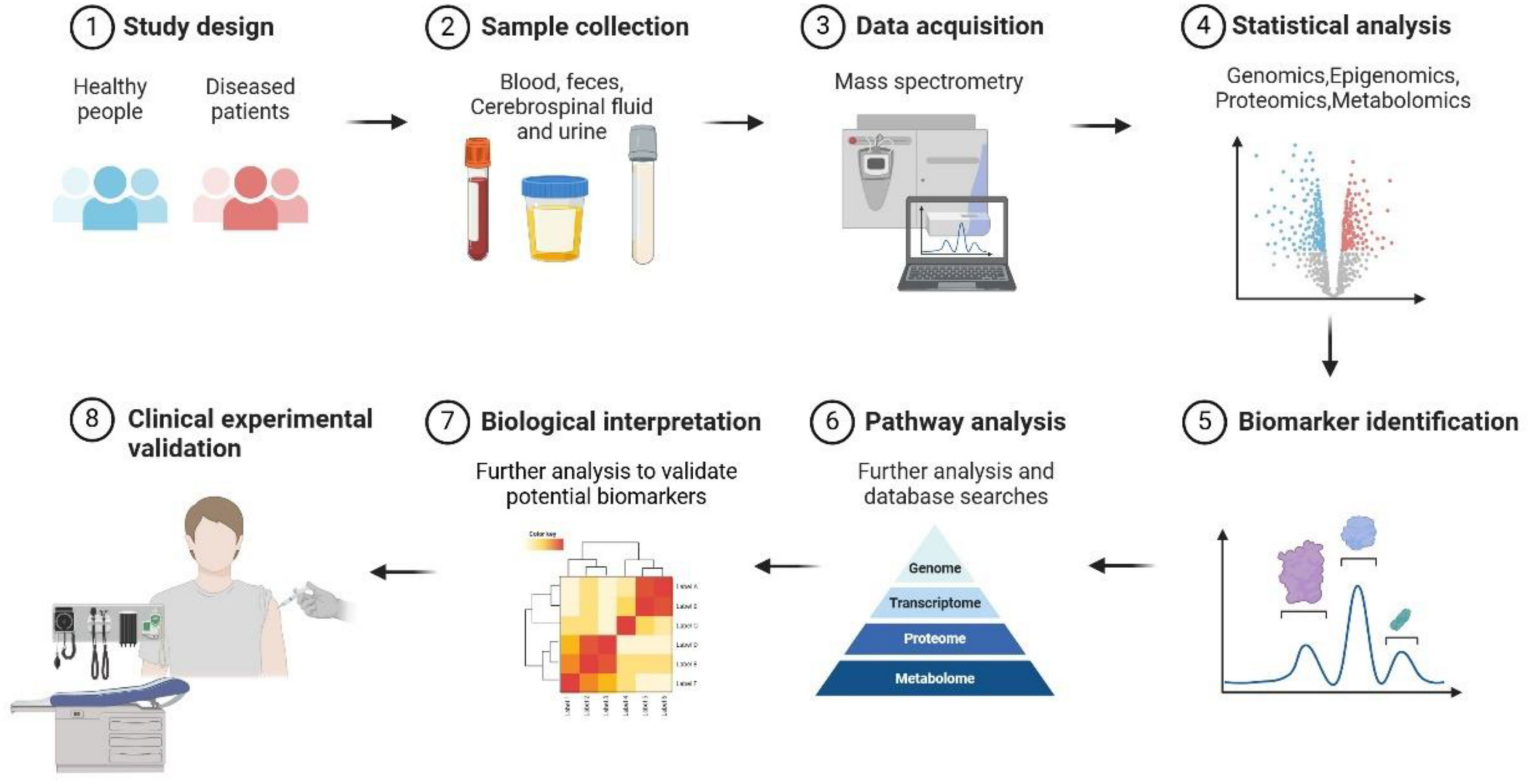
Discovery of biomarkers for ADs through multi-omics. The pathogenesis of ADs is intricate, involving interactions between multi-genetic, multi-pathway and environmental factors. Multi-omics technologies, by integrating multi-dimensional data from genomics, transcriptomics, proteomics, metabolomics and epigenomics, can systematically uncover disease - related molecular characteristics, offering a new perspective for biomarker discovery. This requires a sequence of processes, including experimental design, sample collection, multi-omics research and analysis integration, and ultimately validation through clinical trials.

The advent of multi-omics technologies is redefining biomarker discovery frameworks within contemporary precision medicine. These methodological breakthroughs propel transformative innovations in biological signature identification through systematic integration of pan-omics data - genomics, epigenomics, transcriptomics, proteomics, metabolomics, and microbiome (**Figure 1**). This multi-tiered analytical approach elucidates disease-associated molecular networks that orchestrate pathophysiological progression, thereby transcending the constraints inherent to conventional unidimensional biomarker approaches.

### 3.1 Genomics

Genomics, as the cornerstone of modern medical research, has profoundly reshaped our understanding of the pathogenesis of ADs over the past decade. Genomics analysis, built upon the foundation of bioinformatics knowledge, commonly utilises sequencing technologies such as next-generation sequencing (NGS) and metagenomic sequencing, genotyping and variant detection technologies like genome-wide association studies (GWAS) and gene chips, and gene function analysis technologies including expression quantitative trait loci (eQTL) analysis, chromatin immunoprecipitation sequencing (ChIP-seq), and gene editing (CRISPR-Cas9). Since James Bentham et al. (28) revealed the association between SLE and abnormal regulation of innate immune genes through GWAS, genomics technology has continually advanced, evolving from population genetic associations to single-cell characterisation, cross-omics integration, and causal mechanism dissection. In the study of human ADs, genomics technologies demonstrate dual potential: on the one hand, it can aid in the screening of therapeutic targets, and on the other hand, it can provide biomarkers for disease diagnosis, assessment of disease activity, and monitoring of treatment response.

Identifying new diagnostic and therapeutic biomarkers through genomics. In the exploration of specific biomarkers, the collaborative application of single-cell technologies and bioinformatics tools has become a key breakthrough. Research on LN has found that the significant upregulation of the COL6A3 in renal tissue single-cell transcriptomes (AUC = 0.879) highlights its substantial involvement in the pathogenesis of LN, suggesting its dual value as a diagnostic biomarker and potential therapeutic target (29). AI models based on radiogenomics have revealed the immune regulatory function of COL22A1 in autoimmune encephalitis (AE) associated with glioma, integrating imaging features with genomic variations, which is closely related to the poor prognosis of this disease (30). New proteomic biomarkers, serum osteopontin and interleukin-1(IL-1)-RA, have been identified in children newly diagnosed with type 1 diabetes (T1D) through expression-based GWAS (eGWAS) (31). Moreover, genomic analysis has also identified cathepsin H, interleukin 27 receptor alpha chain (IL27RA), signal regulatory protein gamma, and phosphoglucomutase 1 as potential biomarkers or therapeutic targets for further research in type 1 diabetes (32). By integrating GWAS and eQTL data, researchers have identified the core regulatory role of the ELF1 in SLE renal involvement, with its expression level significantly positively correlated with disease activity (33).

Genomic analysis deepens the understanding of ADs. Through single-cell genomics, new potential drivers of the pathogenesis of systemic juvenile idiopathic arthritis (SJIA) have been identified, including interferon activation, a variety of unique monocyte phenotypes, and platelet activation (9). Quantitative metagenomic studies have revealed that alterations in five types of gut microbiota are associated with the development and progression of ankylosing spondylitis, providing new directions for the development of novel diagnostic tools and potential therapeutic approaches (34). More notably, the breakthrough in serum antisense genomics has enabled the construction of a precise diagnostic model for RA using only peripheral blood samples, successfully distinguishing anti-CCP -positive and negative subtypes and providing an objective molecular basis for clinical classification (31). The results of genetic studies may provide explanations for the phenotypic heterogeneity and biomarkers of drug response in autoimmune hepatitis (AIH). The GWAS of European AIH revealed the strongest association with single nucleotide variants in HLA (35). These findings not only refine the disease classification system but also provide a basis for target selection in personalised treatment.

Genomics aids in uncovering the connections between ADs. SLE and primary Sjögren’s syndrome (pSS) share regulatory networks of transcription factors such as signal transducer and activator of transcription 1 (STAT1) and interferon regulatory factor 7 (IRF7), while the co-expression patterns of genes like interferon-inducible protein 44-like Protein (IFI44L) and interferon-stimulated gene 15 (ISG15) point to a universal activation mechanism of the interferon signalling pathway (36). This discovery of cross-disease commonalities provides a theoretical basis for developing broad-spectrum autoimmune treatment strategies. Similar studies analysing the metagenomic datasets of ADs, including SLE and inflammatory bowel disease (IBD), to identify predictive biomarkers have shown that functional gene analysis (PFAM and CAZymes) indicates a reduction of fibre-degrading enzymes (such as glycosyltransferase 9, glycoside hydrolase 73) and a significant enrichment of short-chain fatty acid (SCFA)-related enzymes (such as pyruvate dehydrogenase, succinate dehydrogenase) in patients with SLE and IBD. Fibre-degrading enzymes and SCFA-related enzymes can serve as potential biomarkers for disease states (37). Fine mapping and functional studies have shown that rs117701653 is a non-coding single nucleotide polymorphism in the CD28/cytotoxic T-lymphocyte-associated protein 4 (CTLA4)/inducible T-cell costimulator (ICOS) locus, a risk variant for RA and T1D (38). Aggelos Banos et al. (39) found that abnormal neutrophil degranulation and excessive B-cell activation constitute unique endotype features distinguishing AAV from SLE, while the higher molecular heterogeneity exhibited by SLE patients may explain their more unpredictable clinical course (39).

### 3.2 Epigenomics

Recent advances in epigenomics have offered novel insights into biomarker discovery and mechanistic exploration in ADs. In the study of diabetic nephropathy (DN) in T1D, a cross-cohort epigenome-wide association study (EWAS) identified 32 differentially methylated CpG sites linked to disease progression (40). Among these, 18 were situated within genes exhibiting differential expression in renal tissue or directly correlated with pathological features, including glomerulosclerosis and interstitial fibrosis. Notably, methylation levels at 21 of these sites independently predicted the risk of end-stage renal failure, underscoring the spatiotemporal specificity of epigenetic regulation in DN (40).

Similarly, investigations into childhood-onset SLE revealed the most pronounced differences in *IFI44L* expression and methylation. Subsequent clinical validation confirmed *IFI44L* methylation as a robust blood-based diagnostic biomarker for childhood-onset SLE, demonstrating an AUC of 0.867, sensitivity of 0.753, and specificity of 1.000 (10).

Research into the pathological mechanisms of MS has demonstrated dysregulation within microRNA (miRNA) networks. Specifically, miR-181a-5p emerges as a critical regulator of neuroinflammation and axonal degeneration through its targeting of three functionally distinct gene groups: *MAP2K1* (modulating the mitogen-activated protein kinase (MAPK) signalling pathway), *CREB1* (governing neuronal survival), and *ATXN1/3* (implicated in cerebellar atrophy) (41). This miRNA’s expression profile has been proposed as a novel epigenetic biomarker for clinical staging in MS (41). Notably, epigenetic plasticity in MS is further evidenced by a monozygotic twin cohort study: discordant DNA methylation patterns between affected twin pairs revealed differential methylation regions (DMRs) at *TMEM232* and *ZBTB16* loci (42). These DMRs not only correlate with disease susceptibility but also mirror glucocorticoid-induced immune remodelling, as demonstrated by longitudinal methylation dynamics in *ZBTB16* (42).

In RA, Gary Craig et al. (43) identified DMRs associated with M1/M2 macrophage polarisation and abnormal proliferation of CD4^+^ T cells through buccal mucosa cell EWAS (e.g., TNFAIP3, IRF5), demonstrating that epigenetic features of non-invasive samples (such as buccal cells) can be used for early diagnosis and risk assessment of RA (43). Moreover, the DNA methylation profile of PBMCs can distinguish responders from non-responders to adalimumab treatment, with the methylation status of genes related to the JAK-STAT pathway (such as *IL6ST*, *SOCS3*) significantly associated with drug responsiveness, providing a predictive tool for personalised treatment (44).

The core role of epigenetic regulation in the common mechanisms of ADs has been further revealed: the majority of DMRs specific to regulatory T cells (Tregs) are contained within Treg-specific super-enhancers and are closely related to the transcription and other epigenetic changes of both naïve and effector Tregs (45). Thus, hypomethylation of CpG sites specific to naïve Tregs plays a key role in regulating the transcription of Treg-specific genes and epigenetic modifications. SNPs within Treg-DMRs can serve as susceptibility markers for ADs (such as T1D, RA, and MS) (45).

Current epigenomics research has driven innovations in the management of ADs from three dimensions: the development of diagnostic biomarkers, in-depth mechanistic dissection, and optimisation of therapeutic strategies. Future efforts need to integrate multi-omics data (such as methylation-transcriptome-proteome) to construct dynamic regulatory models and explore the translational potential of epigenetic editing technologies (such as CRISPR-dCas9) in disease intervention.

### 3.3 Transcriptomics

Systemic autoimmune diseases remain incurable, and robust biomarkers are urgently needed for diagnosis and treatment. Transcriptomic profiling has emerged as a powerful tool for discovery. Mining the Gene Expression Omnibus (GEO), Sasikumar et al. identified eight ferroptosis-related hub genes—NAMPT and SAT1 among them—that distinguish RA with high accuracy (46). Subsequent GEO and RNA sequencing (RNA-seq) studies have uncovered further RA biomarkers: CKAP2 (47), FOXO3 (48), the telomere-associated genes ABCC4, S100A8, VAMP2, PIM2 and ISG20 (49), and the long non-coding RNAs LINC00494, TSPOAP1-AS1, MCM3AP-AS1, LINC01588 and OIP5-AS1 (50), all of which show good diagnostic performance. In pSS-associated interstitial lung disease, CYSLTR1 and SIGLEC1 were found to have diagnostic utility and to nominate montelukast as a potential therapy (51). SLE biomarkers derived from naive and memory B-cell signatures have also been reported (52), while RNA-seq of autoimmune thyroiditis yielded three validated diagnostic genes and two repurposable small molecules (53). Single-cell profiling of autoreactive CD4^+^ T cells in AIH revealed a B-cell-helper signature that may serve as both marker and therapeutic target (54), and a separate AIH study identified a 12-gene set associated with advanced fibrosis (55). CXCL9 shows promise in pSS (56), and a non-invasive pSS model combining labial-gland and peripheral-blood expression profiles with salivary-gland ultrasound has matched the accuracy of labial biopsy (57). In long-standing T1D, differential expression implicates protein post-translational modification, DNA repair and immune-tolerance pathways in protection from vascular complications (58). These findings now require validation in larger, prospective cohorts before clinical implementation.

Biomarkers that forecast therapeutic response or disease activity have also been described. Age-associated B cells regulated by spleen tyrosine kinase can reflect the disease activity of RA (59), and Derlin-1, which is associated with autophagy, has predictive value for the effectiveness of infliximab in treating RA (60). In children with juvenile idiopathic arthritis (JIA), interferon-driven gene signature in PBMCs prior to methotrexate (MTX) therapy identifies those likely to respond well; conversely, patients with low signature expression may require additional agents (61). In paediatric SLE, circular RNAs detected by RNA-seq correlate with disease severity (62), and MX2 serves as an immune-related marker for both diagnosis and activity of SLE (63).

Transcriptomics continues to reveal novel disease subtypes and therapeutic directions. Hubbard et al. validated eight molecular endotypes of lupus identified based on whole blood gene expression. The endotyping of SLE patients based on the transcriptomic profile provides new molecular insights to support individualised treatment management (64). IFN-α-producing monocyte subsets have been identified as candidate biomarkers and therapeutic targets in SLE (11). Neuropsychiatric SLE, which remains clinically challenging, has been shown by Nikolopoulos et al. to potentially benefit from complement inhibitors or B-cell-directed therapies on the basis of whole-blood RNA-seq data (65). RNA-seq of microglia has uncovered differentially expressed genes and circular RNAs implicated in MS pathogenesis, providing a platform for microglia-focused diagnostics and therapeutics (66). Single-cell profiling of early-onset AChR-antibody-positive myasthenia gravis (MG) further identified the migration inhibitory factor–CD74 axis as a putative therapeutic target (67).

Transcriptomics is now uncovering molecular links between ADs and unrelated disorders. Analysis of GEO data has revealed six core genes common to both membranous nephropathy and pan-cancer that may represent shared therapeutic targets (68). Similarly, five genes are co-expressed in SLE and IBD and show robust diagnostic performance (area-under-curve analyses) (69). In addition, analysis of GEO data has revealed shared molecular pathways between RA and osteoarthritis, which may serve as potential diagnostic biomarkers (70).

Bioinformatics can screen for differentially expressed genes through transcriptomic analysis, but distinguishing truly relevant findings from false positives remains a challenge. This limitation can be addressed by validating protein expression levels, uncovering local immune responses, and linking microscopic tissue changes with clinical manifestations. Hai et al. combined transcriptomic screening with immunohistochemistry to identify three oxidative-stress-related genes/proteins as potential biomarkers for thyroid eye disease, offering a route to early detection and risk stratification (71).

### 3.4 Proteomics

Proteomics technology, by systematically analysing the protein expression profiles in body fluids, tissues, and extracellular vesicles (EVs), provides a multi-dimensional perspective for the precise diagnosis, classification, and treatment prediction of ADs. In the field of T1D, plasma proteomics analysis of children with diabetic ketoacidosis (DKA) has revealed that IL1RL1 can serve as a biomarker for inflammatory activity, growth differentiation factor 15 is positively correlated with BMI and HbA1c, indicating its association with metabolic stress and long-term hyperglycaemia, and the abnormal expression of MMP8/MMP9 may predict the risk of neurological complications by mediating blood-brain barrier damage (72). Further research on children with newly diagnosed T1D and partial remission (PR) has uncovered the regulatory potential of proteins such as YWHAZ and SKAP2, which may become potential targets for immune regulation and β-cell protection therapies (73). It is worth noting that although T1D and type 2 diabetes (T2D) share the characteristic of elevated inflammatory protein concentrations (such as IL-6, TNF-α), the inflammatory profile in T2D patients shows a more significant increase, suggesting the heterogeneity in the development pathways of complications between the two (74). In addition, proteomics analysis has identified a potential biomarker for MG, ITIH3, which is significantly elevated in the serum of MG patients during active disease (quantitative MG score > 7) and is positively correlated with quantitative MG and MG-activities of daily living scores (r = 0.399, p < 0.0001) (75).

In the study of MS, comprehensive targeted proteomics analysis has confirmed that serum neurofilament light chain (NfL) is a core differential protein between MS and healthy controls, with its levels directly related to neuroaxonal damage and disease progression (76). To further overcome the limitations of single biomarkers, Tanuja Chitnis et al. (77, 78) used high-throughput platforms (Olink™ and Myriad RBM) to screen 1,400 serum proteins and identified 20 proteins associated with increased clinical and radiological disease activity in MS, with NfL being the strongest biomarker. A multivariate model incorporating NfL (AUROC = 0.813) showed significantly better predictive performance than the univariate model (NfL alone AUROC = 0.794), highlighting the clinical advantages of combined biomarkers. In addition, the abnormal expression of cytokines such as IL-4 and fibroblast growth factor 19 in CSF (73), plasma EV-excitatory amino acid transporter 2 (79), and the predictive ability of baseline GFAP/FLRT2 for choroid plexus volume expansion (80) provide new dimensions for early warning of MS (73, 79, 80). Another study identified CD138 as a specific CSF biomarker for MS, with an AUC of 0.85 (95% CI 0.75-0.95) (81). In neuromyelitis optica, proteomics analysis of CSF found that combined detection of CSF/serum neural cell adhesion molecule 1 and serum somatostatin effectively improved the diagnostic accuracy of aquaporin 4 antibody-negative cases (82).

Proteomic studies of LN focus on urine samples. Kazuoto Hiramoto et al. (83) identified urine calgranulin B, MCP-1, and IGFBP-5 as predictors of histological severity through screening 1,305 proteins. Yaxi Li et al. (84, 85) expanded the urinary biomarker spectrum (e.g. ICAM-2, FABP4, FASLG, IGFBP-2, SELE, TNFSF13B/BAFF) and identified novel kidney injury-related proteins such as TECK, TSLP, PDGFRα, and MDC. In primary immune thrombocytopenia (ITP), Olink precision proteomics found that the combination of inflammatory proteins such as CXCL11 and TGF-β1 and the detection of MMP-9/THBS1 (AUC = 0.87) significantly improved diagnostic specificity. Proteins such as CFL1 and APOA1/GC/TF were associated with splenectomy response and drug treatment prognosis, respectively (86–89).

Regarding treatment-response prediction, RA studies highlight proteomics’ translational potential. Ara Cho et al. used high-precision proteomics to identify protein biomarkers that can predict the clinical response of RA patients to Tocilizumab (TCZ) (90). The TCZ treatment response prediction model (a combination of 13 proteins including CD5L, ICOSLG, HP, AGT, CRP, FN1, F13B, CFHR2, LYZ, BASP1, APOF, CD163, IL1RAP) achieved 100% sensitivity with an AUC of 0.84 (90). Single-cell proteomics technology revealed that the frequency of PD-1-positive memory B cells and plasmablasts was negatively correlated with CRP, providing a basis for immune checkpoint intervention (90, 91). In the study of giant cell arteritis (GCA), the random forest model achieved an accuracy rate of 95% for active disease and 98.3% for inactive disease, with LAMA3 being a potential biomarker for disease activity (92). Proteomic analysis also identified three proteomic subgroups in GCA (93), finding that the protective effects of metabolic-related biomarkers such as FBP1 and the associations with macrophage activation (adhesion G protein-coupled receptor E2 and metrnl) and pro-inflammatory signals (ROR1) had protective effects on the development of GCA (94).

Moreover, circulating EVs’ protein composition has expanded biomarker sources. The specific EV proteins of Sjögren’s syndrome (SjS) and the abnormal expression of von Willebrand factor/insulin receptor in obstetric antiphospholipid syndrome have opened up new avenues for non-invasive detection (95, 96).

### 3.5 Metabolomics

Metabolomics technology, by systematically analysing the changes in endogenous metabolites within organisms, offers a new perspective for the precise diagnosis and treatment of ADs. In SLE, the metabolic response characteristics of mesenchymal stem cell transplantation (MSCT) treatment show that the change in thiamine monophosphate levels is the strongest predictor of treatment success, with a 35% increase in its levels significantly associated with treatment response, indicating that it could serve as a key biomarker for predicting the efficacy of MSCT (12). Further research has found that a combined model of plasma amino acid metabolites (histidine, lysine, tryptophan) exhibits perfect diagnostic performance (AUC = 1.0, with sensitivity, specificity, and accuracy all at 100%) in distinguishing LN from SLE, providing a new strategy for the non-invasive classification of LN (14).

Metabolomics studies on RA have revealed the molecular basis of the heterogeneity in treatment responses. The metabolic characteristics of responders to methotrexate, TNF inhibitors, and IL-6 inhibitors differ mainly in the tricarboxylic acid cycle, amino acid, and lipid metabolism pathways, offering potential predictive indicators for personalised medication (97). Notably, metabolomics has also provided insights into the metabolic regulatory mechanisms of traditional Chinese ethnic medicines (such as Mubieji Granules (MBm) and Longzuantongbi Granules (LZTBG)) in treating RA (98). The research shows that although both share some anti-inflammatory metabolites (such as linoleic acid derivatives), their region-specific components may exert effects through unique pathways such as sphingolipid metabolism (98).

In MS, metabolomics dissects the disease progression mechanisms from multiple dimensions. A combined model of plasma structural lipids (phosphatidylcholine, phosphatidylethanolamine) and NfL can predict axonal damage, while polyunsaturated fatty acids and their derivatives have a significant protective effect on disease activity (q < 0.001) (99). Comparative analysis of CSF and serum further reveals gender/age-specific differences in the metabolism of endocannabinoids and glucocorticoids, providing a basis for the heterogeneous management of MS (100). In addition, serum serine levels determined by genetics have been shown to have a causal association with MS risk, opening up new directions for the selection of intervention targets (101).

Non-targeted metabolomics studies on MG have used machine learning to identify five biomarkers: behenic acid (AUC = 0.944), uridine diphosphate-N-acetylglucosamine (AUC = 0.951), arachidonic acid (AUC = 0.951), β-glycerophosphate (AUC = 0.933), and L-asparagine (AUC = 0.877) (102). Their abnormal expression is closely related to immune attack at the neuromuscular junction (102). In antiphospholipid syndrome, Zhaoer Yu et al. (103) used non-targeted lipidomics to find that the combination of phosphatidylcholine and acylcarnitine significantly improved diagnostic specificity with an AUC of 0.865.

Metabolomics has also expanded the understanding of the pathological mechanisms of AE. By analysing the metabolic profiles of CSF in patients with AE, it was found that the biosynthesis of unsaturated fatty acids and the metabolism of α-linolenic acid are disrupted, which helps elucidate the pathophysiological mechanisms of AE (104). Using a two-sample mendelian randomisation (MR) method, causal relationships between plasma metabolites and five common ADs (IBD, MS, T1D, SLE, and RA) were established, revealing multiple metabolic pathways associated with ADs (105). Bone marrow biopsy samples from patients with ITP were subjected to metabolomics analysis, and machine learning algorithms were used to identify novel biomarkers that can predict treatment responses in ITP patients, including the biosynthesis of long-chain fatty acids, oxidised lipids, glycerophospholipids, phosphatidylcholine, and phosphatidylethanolamine (106). In sequential sera from patients with GCA and polymyalgia rheumatica, metabolomics based on nuclear magnetic resonance (combined with N-acetylglycoproteins and choline from phospholipids) can distinguish between active and inactive disease (107).

The application scenarios of metabolomics have expanded from blood to other body fluids such as saliva and urine: salivary metabolite biomarkers of SjS (alanine, isovaleric acid, succinic acid) show high sensitivity (0.750-1.000) and specificity (0.615-0.692), while urinary metabolites in RA patients such as isobutyric acid (AUC = 0.83), dimethylglycine (AUC = 0.65), and 2-oxoisovaleric acid (AUC = 0.49) provide new means for early warning of skeletal muscle atrophy (108, 109). Metabolomics has also explored the metabolic profiles of DKA in T1D, achieving 100% accurate distinction between severe DKA and insulin-controlled states through dynamic changes in the spectra of ketone bodies and phosphatidylcholine (110).

By dissecting the dynamic changes in metabolic networks from multiple dimensions, metabolomics not only provides highly sensitive combinations of biomarkers for ADs but also demonstrates unique advantages in disease classification, prediction of treatment responses, and elucidation of pathological mechanisms, laying an important foundation for the development of personalised diagnostic and therapeutic strategies.

### 3.6 Microbiome

In recent years, significant progress has been made in the study of the association between the gut microbiome and ADs. A substantial amount of evidence indicates that the ecological imbalance of gut microbiota is closely related to the pathogenesis and clinical manifestations of various ADs. Through advanced techniques such as high-throughput sequencing and machine learning, researchers have identified microbiota markers with diagnostic value in different ADs and have explored the potential mechanisms of microbiota-host interactions in depth.

In the study of SLE, 18 key bacterial markers were identified from urine microbiome analysis using 16S rRNA sequencing technology. Random forest algorithm was employed to select key genera that could accurately distinguish between healthy controls, SLE patients without LN, and SLE patients with LN. The diagnostic model constructed showed perfect discrimination ability between LN patients and healthy controls (AUC = 100%) (13). By using machine learning algorithms in combination with significantly dysregulated microbiota for the early identification of SLE patients, four machine learning algorithms were applied, namely logistic regression (LR), adaptive boosting (AdaBoost), random forest, and extreme gradient boosting (XGBoost). Among them, XGBoost performed the best, with accuracy, sensitivity, specificity, positive predictive value, negative predictive value, and area under the receiver operating AUC reaching 0.844, 0.750, 0.938, 0.923, 0.790, and 0.930, respectively (15). Similarly, in JIA research, an XGBoost model constructed based on 10 faecal microbiota markers selected by LASSO regression demonstrated excellent diagnostic performance, with an AUC of 0.976 and an accuracy of 0.914 (111). These findings not only provide new biomarkers for disease diagnosis but also reveal the commonalities and differences in microbial signatures among different ADs. For instance, both IBD and SLE patients exhibit an enrichment of the genus *Streptococcus*, while healthy individuals are dominated by the genera *Anaerostipes* and *Saccharofermentans* (37).

Further research has revealed that the gut microbiota is involved in immune regulation through various pathways. In patients with RA, the abundance of specific microbes is negatively correlated with CD28 expression, profoundly demonstrating the complex interplay between the gut microbiota and RA-related immune regulation mechanisms (112). Meanwhile, newly diagnosed RA patients show a reduction in Treg and follicular helper T cells, which are associated with changes in the gut microbiota and their metabolites, involving Ruminococcus 2, (involved in the biosynthesis pathway of unsaturated fatty acids), and 3-methoxyindole (involved in the tryptophan metabolism pathway) (113). In patients with MG, the community characteristics of the gut microbiota, analysed by 16S rRNA gene sequencing, show a reduction in the genus *Clostridium* and a decrease in short-chain fatty acid levels, which may be related to the immune disorder at the neuromuscular junction (114). These findings provide important clues for understanding the regulatory mechanisms of the microbiota-immune-metabolism axis.

It is worth noting that the technical methods in microbiome research are also constantly innovating. The microbiome network flow entropy (mNFE) technique can identify the key pre-disease state based on a single faecal sample. In the study of T1D, it successfully predicted serum conversion. This technique can sensitively detect the key pre-disease state before the sudden change of T1D at different taxonomic levels. This means that mNFE can robustly and efficiently detect the pre-disease state and identify the corresponding dynamic network biomarkers through a single faecal sample from each individual (115). PICRUST2 functional prediction revealed 21 microbial genera associated with T1D, with AUC values of 0.962 and 0.745 in the discovery and validation sets, respectively (116). These technological advancements offer new possibilities for early disease warning and intervention. In addition, Malin Bélteky et al. pointed out through random forest analysis that the composition of infant gut microbiota is associated with the occurrence of T1D (117). The genus Ruminococcus is an important determinant distinguishing control infants from those who will develop T1D in the future. The genera *Alistipes* (more abundant in control infants) and *Fusicatenibacter* (with different abundance patterns in case and control infants) are key factors distinguishing infants who will develop T1D in the future (117).

The research has also expanded to other sample types and diseases. In patients with SjS, changes in the oral microbiome, such as a decrease in *Lactobacillus* and an increase in *Streptococcus*, are associated with salivary gland dysfunction. This suggests that the oral microbiome could potentially serve as a diagnostic biomarker for SjS (118). IgA nephropathy patients exhibit cross-site (oral-pharyngeal-gut-urine) microbial dysregulation, with an increase in the genera *Bergeyella* and *Capnocytophaga* in the oral cavity being of diagnostic value. Using a random forest classifier to predict IgAN, the best accuracy in the discovery phase reached 0.879, and in the validation phase, it was 0.780 (119). These findings broaden our understanding of the role of mucosal microbiota in ADs. In terms of kidney diseases, the study of microbial communities in patients with idiopathic membranous nephropathy (IMN) employed 16S rRNA gene sequencing technology and used Tax4Fun to predict their functional characteristics (120). Twenty characteristic microbial biomarkers were successfully identified, and the disease prediction model constructed had a diagnostic accuracy of up to 93.53%, providing important clues for a deeper understanding of the pathogenesis of IMN (120). For patients with relapsing-remitting MS (RRMS), the differential bacterial genera in the gut microbiota were included in LR analysis. It was found that the genera *Gemella* (with an AUC of 75.0 and a confidence interval of 60.6 - 89.4) and *Bilophila* (with an AUC of 70.2 and a confidence interval of 50.1 - 90.4) had the best fit. These genera may have important predictive roles in the prognosis and diagnosis of RRMS (121).

In summary, the study of the gut and other microbiota has provided a new perspective for understanding the pathogenesis of ADs and has developed new biomarkers for disease diagnosis and prognostic assessment. Significant achievements have been made in the identification of disease-specific microbial biomarkers, the construction of efficient diagnostic models using machine learning and other technologies, and the elucidation of the interplay between microbial communities and immune regulation mechanisms. Future research needs to further verify the specificity of these microbial biomarkers and explore individualised treatment strategies based on microbial interventions to promote the translation of this field into clinical applications.

### 3.7 Multi-omics integration strategies

Network Analysis graph neural networks (GNN) integrates multi-omics data to construct the “gene-metabolite-immune cell” interaction network in RA patients, identifying key node biomarkers. Encompassing genomics, transcriptomics, proteomics, and metabolomics, this approach provides a powerful tool for comprehensively analysing molecular changes in biological systems. Multi-omics research not only elucidates pathological mechanisms but is also crucial for identifying new diagnostic biomarkers and therapeutic targets. Treatment strategies for ADs require the integration of data from multiple aspects to enhance therapeutic precision. Omics data offer insights at the molecular level, while integration with clinical data provides biological relevance to these molecular discoveries.

#### 3.7.1 Multi-omics biomarker screening and the clinical application of joint models

By integrating data from transcriptomics, proteomics, and metabolomics, highly diagnostic biomarker combinations have been successfully identified. For instance, in autoimmune thyroid disease (AITD), a serum joint model comprising ISG15 (an interferon-induced protein specific to hyperthyroidism), ZNF683 (a T-cell differentiation regulator associated with hypothyroidism), and IGHG3 (a B-cell activation marker) demonstrated excellent diagnostic performance (AUC > 0.85) (122). The expression levels of these biomarkers were significantly correlated with clinical indicators (such as TRAb and TSH), and their clinical application potential was validated through decision curve analysis (122). A similar strategy was extended to thyroid eye disease, where the joint model based on ISG15, ZNF683, and IGHG3 showed good diagnostic efficacy for both hyperthyroidism and hypothyroidism (AUC > 0.85). The combination of tear protein biomarkers S100A4 (fibroblast activation) and PIP (adipogenesis regulation) can predict disease severity, promoting precise intervention (123). Additionally, through the integrated analysis of transcriptomics and proteomics, it was found that MS patients develop immunogenicity to IFN-β treatment. A joint model of STAT1, CCL2, and CD79B predicted the production of anti-drug antibodies with a sensitivity of 85% and specificity of 88% (124). This study also clarified the association between immune system dysfunction and blood-brain barrier damage with the severity of MS and proposed three biomarkers related to MS risk: CD40, AHSG, and FCRL3 (125). By integrating gut microbiome and blood metabolomics data, a biomarker panel has been identified that can distinguish acute MS from chronic MS. This panel comprises four bacterial genera (three decreased: *Lysinibacillus*, *Parabacteroides*, *UBA1819*; one increased: *Lachnospiraceae*) and two glycerophospholipid metabolism-related metabolites (one increased: phosphatidylethanolamine; one decreased: cardiolipin). This combination of biomarkers effectively differentiates patients with chronic MS from those with acute MS (126).

In a multi-centre study of T1D, two potential factors significantly associated with β-cell function decline were identified through the integration of transcriptomics, miRNA, proteomics, metabolomics, and immunomics data: Factor 15 and Factor 18, both of which were negatively correlated with rapid β-cell function loss (Factor 15: standardised β = -0.31, *p* = 0.0018; Factor 18: standardised β = -0.37, *p* = 0.00018) (127).

#### 3.7.2 Cross-omics dissection of disease mechanisms: metabolism-immunity interactions and pathway regulation

Multi-omics technologies reveal the synergistic effects of metabolic reprogramming and immune dysregulation in ADs. In the multi-omics analysis of metabolic diseases in RA patients (RA_MD) (16S rRNA and internal transcribed spacer (ITS) gene sequencing, metabolomics, transcriptomics, proteomics, and phosphoproteomics), lipid metabolism disorders (such as the accumulation of 1-palmitoyl-sn-glycero-3-phosphocholine) were identified as core features. A LASSO model based on 17 metabolites could precisely distinguish RA_MD from RA alone (AUC = 0.958), indicating the driving role of the metabolic microenvironment in extra-articular complications (128). In SLE, the integration of single-cell RNA sequencing and proteomics revealed that interferon signalling pathways (such as STAT1, MX1, etc.) and mitochondrial autophagy-related proteins (PHACTR2, MAP2K1, etc.) are involved in disease diagnosis and worsening prediction (with an AUC as high as 0.990), providing a basis for stratified treatment (129).

#### 3.7.3 Multi-omics decoding of traditional Chinese medicine mechanisms: multi-target regulatory networks

Multi-omics technologies offer a new perspective for elucidating the molecular mechanisms of traditional Chinese medicine formulas. Qingre Huoxue Decoction (QRHXD), a widely used traditional Chinese medicine formula in clinical practice, has shown significant efficacy in treating RA. Fuyuan Zhang et al. (130) used proteomics and metabolomics to jointly analyse that QRHXD activates the AMPK pathway by inhibiting the rate-limiting enzyme of gluconeogenesis, FBP1, thereby reducing RA activity. Metabolomics and network pharmacology analysis of Simiao Pill (SMW) revealed that it alleviates the fibrotic process of RA-associated interstitial lung disease (RA-ILD) by regulating ferroptosis-related metabolites (such as glutathione peroxidase 4) (131). In addition, combining serum pharmacochemistry and metabolomics, it was found that shaoyao gancao fuzi decoction regulates the arachidonic acid-prostaglandin pathway (AA-PGH2-PGE2/PGF2α) to inhibit pyroptosis of synovial cells and alleviate joint symptoms (132).

#### 3.7.4 Mendelian randomisation and causal target discovery

MR combined with multi-omics data accelerates the identification of causal genes and therapeutic targets for diseases. In SLE, MR and HEIDI tools were applied to comprehensively analyse large-scale GWAS data and eQTL data, identifying seven new potential functional genes associated with SLE, including BLK, ELF1, STIM1, B3GALT6, APOLD1, INPP5B, and FHL3. Based on multi-omics and experimental validation of their correlation with disease activity, IFITM3 was identified as a diagnostic biomarker (133). In MS, Wei Yang et al. (134) successfully determined EVI5, OGA, and TNFRSF14 as potential therapeutic targets by integrating MR analysis and Bayesian colocalisation analysis, laying the foundation for the design of new immunotherapies. In GCA, MR analysis was conducted on plasma proteins, blood-urine biomarkers, and metabolites, revealing causal associations between DDIT4 and ARHGAP15 and elucidating the molecular mechanisms of abnormal activation of CD4^+^ memory T cells (135).

#### 3.7.5 Cross-disease common mechanisms

Multi-omics reveal shared pathological features of ADs. For example, integrated multi-omics analysis showed that RA and colorectal cancer share the MYO9A gene, highlighting its clinical significance of cross-organ immune-metabolic interactions and also serving as an immune-related therapeutic target (136). A multi-omics analysis of the treatment response to ocrelizumab in RRMS patients indicated that the compensatory increase in BAFF after CD20 depletion promotes B-cell reconstitution, providing a basis for optimising targeted therapies (137).

Multi-omics technologies, through multi-dimensional integration (gene-protein-metabolite) and causal inference (MR), have reshaped the study of ADs. Joint models of cross-omics biomarkers (such as the AITD triad) significantly enhance diagnostic specificity and clinical utility; from metabolism-immunity interactions (RA_MD) to interferon signal abnormalities, the root causes of disease heterogeneity are systematically dissected; multi-target regulatory mechanisms of traditional Chinese medicine (such as QRHXD, SMW) and MR-identified new targets (EVI5, BLK) offer a dual-track strategy for personalised treatment. Future efforts need to break through data integration bottlenecks (such as spatiotemporal alignment of single-cell and bulk omics) and promote translational loops (clinical validation of biomarkers-targets-interventions) to achieve a leap from “molecular atlas” to “precision intervention”.

In summary, the development of multi-omics technologies has facilitated the discovery of biomarkers for the diagnosis and treatment of ADs and deepened our understanding of these challenging conditions (**Table 2**). However, a significant challenge in applying omics technologies is managing and interpreting complex biological information in large-scale datasets. Therefore, it is essential to incorporate systems biology strategies and utilise advanced modelling tools to address the variability, complexity, and non-linearity of biological interactions (138).

**Table 2:** Discovery of biomarkers for autoimmune diseases driven by multi-omics.

Autoimmune diseases	Omics	Novel and potential biomarkers
SLE	Genomics Epigenomics Transcriptomics Metabolomics Microbiome Multi-omics	ELF1 (33), STAT1 (36), IRF7 (36), IFI44L (36), ISG15 (36), PFAM (37), CAZymes (37), glycosyltransferase 9 (37), glycoside hydrolase 73 (37), pyruvate dehydrogenase (37), succinate dehydrogenase (37), hub genes associated with B cells (IFI27、IFITM1、MX2、IRF7) (52), circular RNAs (hsa_circ_0017675, hsa_circ_0013503, hsa_circ_0013852, hsa_circ_0003563, hsa_circ_0000612, hsa_circ_0011482, hsa_circ_0003621, hsa_circ_0019518, hsa_circ_0008277, hsa_circ_0008898) (62),MX2 (63), (IL-6 signalling, type-I interferon) (Neuropsychiatric SLE) (65), (KLRF1、GZMK、KLRB1、CD40LG、IL-7R) (69), thiamine monophosphate levels (12), amino acid metabolites (histidine, lysine, tryptophan) (14), 18 key bacterial markers (13), *Streptococcus* (37), (STAT1, MX1, PHACTR2, MAP2K1) (129), IFITM3 (133).
Rheumatoid Arthritis (RA)	Genomics Epigenomics Transcriptomics Proteomics Metabolomics Microbiome Multi-omics	31, SNP rs117701653 (38), TNFAIP3 (43), IRF5 (43), NAMPT (46), SAT1 (46), CKAP2 (47), FOXO3 (48), (ABCC4, S100A8, VAMP2, PIM2 and ISG20) (49), lncRNAs (LINC00494, TSPOAP1-AS1, MCM3AP-AS1, LINC01588 and OIP5-AS1) (50), age-associated B cells (59), Derlin-1 (60), a combination (CD5L, ICOSLG, HP, AGT, CRP, FN1, F13B, CFHR2, LYZ, BASP1, APOF, CD163, IL1RAP) (90), (homocysteine, glycerol-3-phosphate, and diphosphoglyceric acid, N-acetylglucosamine, N-acetylgalactosamine, and N-acetylneuraminic acid) (97), urinary metabolites (isobutyric acid, dimethylglycine, 2-oxoisovaleric acid) (108, 109),113, *Ruminococcus* 2 (113), FBP1 (130), MYO9A (136).
Lupus Nephritis (LN)	Genomics Proteomics Metabolomics	COL6A3 (29), (urine calgranulin B, MCP-1, and IGFBP-5) (83), plasma amino acid metabolites (histidine, lysine, tryptophan) (14).
Type 1 Diabetes (T1D)	Genomics Epigenomics Proteomics Microbiome	Osteopontin (31), IL-1-RA (31), SNP rs117701653 (38),41,59, YWHAZ (73), SKAP2 (73), ketone bodies (110), phosphatidylcholine (110), mNFE (115), *Alistipes* (117), *Fusicatenibacter* (117).
Primary Sjögren’s Syndrome (pSS)	Genomics Transcriptomics	STAT1 (36), IRF7 (36), IFI44L (36), ISG15 (36), CYSLTR1 (51), SIGLEC1 (51), CXCL9 (56).
Inflammatory Bowel Disease (IBD)	Genomics Microbiome	PFAM (37), CAZymes (37), glycosyltransferase 9 (37), glycoside hydrolase 73 (37),70, *Anaerostipes* (37), *Saccharofermentans* (37).
Autoimmune Encephalitis (AE)	Genomics Metabolomics	COL22A1 (30), α-linolenic acid (104).
Juvenile Idiopathic Arthritis (JIA)	Genomics Transcriptomics Microbiome	IFN activation program (9)(SJIA), IFN-driven genes (61), 10 faecal microbe biomarkers (Firmicutes, Bacteroidota, Proteobacteria, Faecalibacterium, Alloprevotella, UCG-002, Dialister, Lachnoclostridium, Monoglobus and Veillonella) (111).
Multiple Sclerosis (MS) (RRMS)	Epigenomics Transcriptomics Proteomics Metabolomics Microbiome	MiR-181a-5p (41), DMRs at TMEM232 and ZBTB16 loci (42),67, NfL (76), IL-4 (73), FGF-19 (73), EV-EAAT2 (79), CD138 (81), plasma structural lipids (phosphatidylcholine, phosphatidylethanolamine) and polyunsaturated fatty acids (99), *Gemella* and *Bilophila* (RRMS) (121), STAT1 (124), CCL2 (124), CD79B (124), CD40 (125), AHSG (125), FCRL3 (125), a panel (four bacterial genera (Lysinibacillus, Parabacteroides, UBA1819, Lachnospiraceae) and two glycerophospholipid metabolism-related metabolites (phosphatidylethanolamine, cardiolipin)) (126), EVI5 (134), OGA (134), TNFRSF14 (134).
Autoimmune Hepatitis (AIH)	Genomics Transcriptomics	Single nucleotide variants (SNVs) in HLA: DRB1*03:01, DRB1*04:01(Europeans); DRB1*04:04, DRB1*04:05, DRB1*13:01 (Latin Americans); DRB1*04:01 and DRB1*04:05 (Japanese) (35), CD4 T cell subset(PD-1, TIGIT, HLA-DR) (54), 12 genes associated with advanced fibrosis (ATP5F1B*, SNX3, SLC2A1*, TMCC2, CREG1, TNIP1, ZMAT2, HK1, PTMS*, TMEM183A, CTSB, TMEM158) (55).
Immune Thrombocytopenia (ITP)	Proteomics Metabolomics	(CXCL11, TGF-β1, MMP-9/THBS1, CFL1, APOA1/GC/TF) (86–89), (long-chain fatty acids, oxidised lipids, glycerophospholipids, phosphatidylcholine, phosphatidylethanolamine) (106).
Sjögren’s Syndrome (SjS)	Proteomics Metabolomics Microbiome	von Willebrand factor/insulin receptor (95, 96), (alanine, isovaleric acid, succinic acid) (108, 109), (*Lactobacillus*, *Streptococcus*) (118),
Myasthenia Gravis (MG)	Transcriptomics Proteomics Metabolomics Microbiome	CD74 (67), ITIH3 (75), (behenic acid, uridine diphosphate-N-acetylglucosamine, arachidonic acid, β-glycerophosphate, and L-asparagine) (102), *Clostridium* (114).
Giant Cell Arteritis (GCA)	Proteomics Metabolomics	LAMA3 (92), (FBP1, ADGRE2, metrnl, ROR1) (94), (N-acetylglycoproteins, choline) (107).
Idiopathic Membranous Nephropathy (IMN)	Microbiome	Citrobacter (120), Akkermansia (120).
Autoimmune Thyroid Disease (AITD)	Proteomics Transcriptomics	ISG15 (122), ZNF683 (122), IGHG3 (122).
Thyroid Eye Disease	Multi-Omics	(ISG15, ZNF683, IGHG3, S100A4, PIP) (123).

## 4 Discussion and outlook

**Figure 2:**
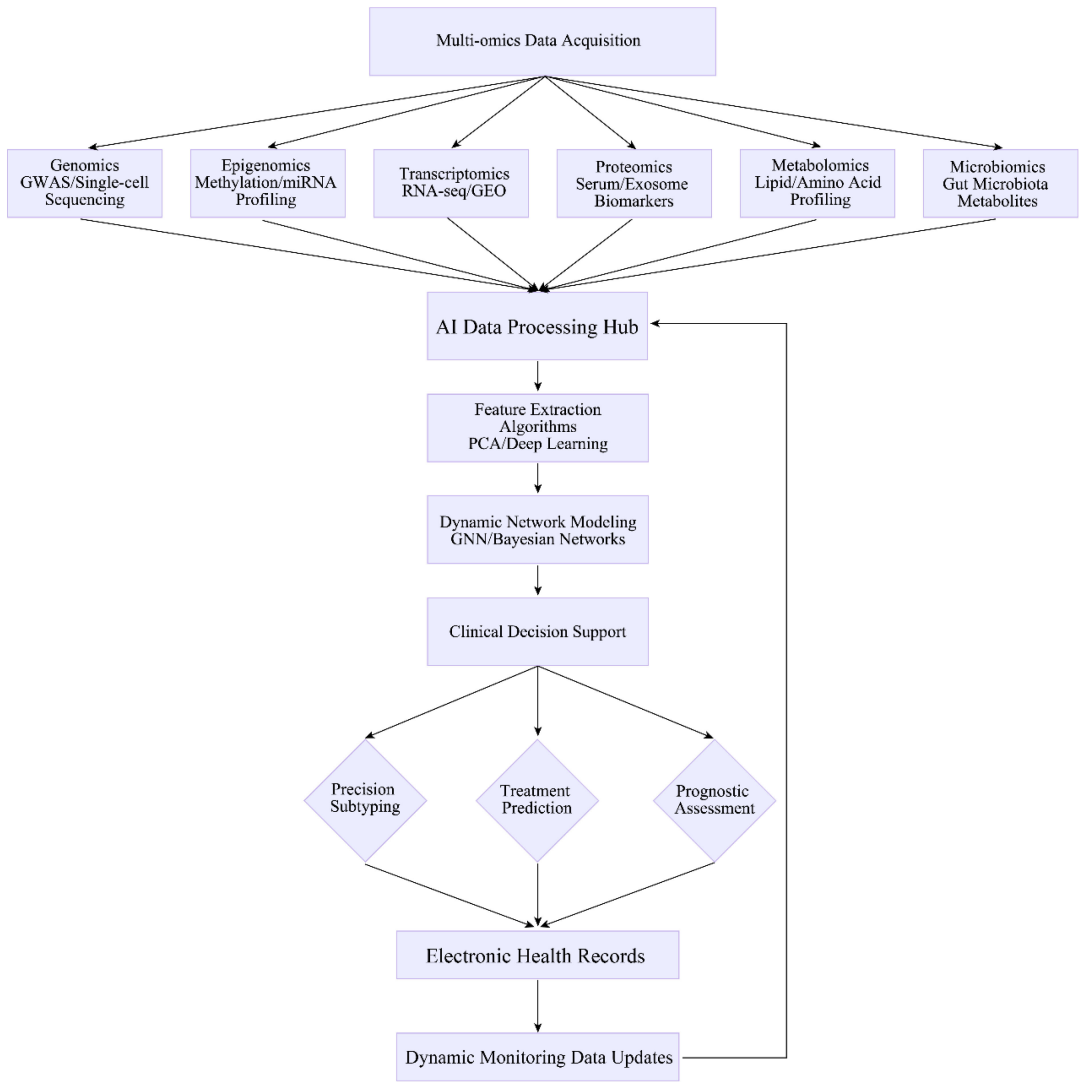
The “multi-omics - dynamic monitoring - clinical decision-making” closed-loop model. Multi-Omics Data Integration Layer: Integrates multi-dimensional datasets covering genomics, transcriptomics, proteomics, metabolomics, and epigenomics to build a comprehensive disease landscape. Dynamic Monitoring Layer: Monitors multiple biomarkers to track their dynamic temporal changes during therapeutic interventions. Clinical Decision Optimisation Layer: Maps multi-dimensional omics data to the clinical phenotype space via machine learning models to generate treatment recommendations with clear action orientation. The system forms a continuously optimised knowledge loop through a continuous learning mechanism.

We propose the establishment of a “Multi-Omics - Dynamic Monitoring - Clinical Decision” closed-loop framework (**Figure 2**), which will systematically address the translational gap between biomarker discovery and clinical implementation. By integrating multi-dimensional biological data, capturing temporal biological dynamics, and enabling evidence-based clinical optimisation, this model establishes a complete translational pathway from molecular mechanism elucidation to therapeutic intervention. Its principal innovation resides in creating an iterative interface between experimental research and personalised clinical practice, ensuring continuous validation of molecular insights through real-world therapeutic outcomes.

Multi-omics data integration layer: This stratum integrates multi-dimensional datasets spanning genomics, transcriptomics, proteomics, metabolomics, and epigenomics, enabling comprehensive disease mapping while uncovering cross-scale regulatory mechanisms inaccessible to single-omics approaches. A representative study synthesised 16S rRNA/ITS sequencing, metabolomic, transcriptomic, proteomic, and phosphoproteomic profiles in RA patients with metabolic comorbidities (RA_MD), identifying lipid metabolism dysregulation as a pathognomonic feature. A LASSO regression model incorporating 17 differential metabolites (e.g. 1-palmitoyl-sn-glycero-3-phosphocholine, hexadecanoic acid) demonstrated exceptional diagnostic discrimination (AUC = 0.958) between RA_MD and RA cohorts (128).
Dynamic monitoring layer: Employing scRNA-seq, this component tracks temporal immune cell subset dynamics during therapeutic interventions. Parallel exosomal profiling facilitates non-invasive surveillance, exemplified by serum exosomal miRNA trajectory mapping in sepsis management. This dual-modality approach captures both cellular heterogeneity and systemic biomarker flux.
Clinical decision optimisation layer: This stratum employs machine learning architectures (e.g. hybrid architecture of support vector machines and GNN) to project multi-dimensional omics signatures onto clinical phenotype spaces, yielding actionable therapeutic inferences. Exemplifying this approach, predictive modelling of MS progression achieved robust stratification of long-term disability risk (AUC = 0.811) through integration of multi-omics biomarkers (139). Crucially, the system incorporates an auto-iterative mechanism that systematically assimilates real-world therapeutic feedback - including pharmacotherapeutic adjustments and radiologically quantified disease trajectory metrics from electronic health records (EHR)-into the multi-omics repository, thereby establishing a self-optimising translational circuit.

While this framework appears to solve the problem of translating multi-omics discoveries into clinical applications in theory, it is merely an idealised concept and is far from being practically applied. There are severe challenges in transforming theoretical blueprints into clinical realities. The first is the huge gap in technical integration and data processing. High-dimensional data from different omics levels are highly heterogeneous, with essential differences in dimensions, scales, noise and formats. Due to the difficulties in data standardisation and normalisation, they cannot be integrated together and there is a need to develop advanced computational tools. There is a fundamental contradiction between computational modelling and clinical interpretability. The framework relies on complex machine learning models. Although some models may have good predictive performance, their decision-making processes are often inexplicable. The risk of overfitting in high-dimensional models poses statistical challenges in obtaining robust and clinically valuable insights. The last key barrier is the feasibility of clinical implementation. Its feasibility must be rigorously validated in large-scale prospective clinical trials before clinical application. Multi-omics analysis is currently extremely costly, and there is confusion over whether the huge costs can bring corresponding clinical benefits that cannot be achieved by existing routine examinations. The process of collecting, processing, sequencing and analysing multi-omics samples is lengthy and cannot meet the timeliness required in real clinical practice. Moreover, it is necessary to establish the infrastructure for storing and managing massive amounts of data, and there are also key ethical and legal issues.

The deep integration of multi-omics technologies and AI is reshaping the paradigm of diagnosis and treatment for ADs. By integrating multi-dimensional data from genomics, epigenomics, proteomics, metabolomics, microbiome, and combining cutting-edge technologies such as single-cell sequencing and exosome analysis, researchers have successfully revealed the molecular basis of disease heterogeneity and developed highly sensitive joint biomarker models (such as the 17-metabolite model for RA_MD with an AUC of 0.958 (128)). Network analysis (such as GNN) has enabled dynamic dissection of the “gene-metabolite-immune” interaction network, while the “multi-omics - dynamic monitoring - clinical decision-making” closed-loop model has promoted the closed-loop optimisation from basic research to clinical translation. The integration of the digital phenome (such as data from wearable devices) with traditional omics has further expanded the spatiotemporal dimensions of disease dynamic monitoring.

In the future, the synergy between multi-omics and AI will accelerate the implementation of personalised diagnosis and treatment: targeted interventions based on metabolism-immunity interaction networks, causal target development based on MR, and precise dissection of the multi-target regulatory mechanisms of traditional Chinese medicine formulas are expected to break through existing therapeutic bottlenecks. However, technological translation still faces multiple challenges: standardised integration of multi-source data (such as spatiotemporal alignment of single-cell and bulk omics), interpretability of AI models (such as the trust gap between black-box algorithms and clinical decision-making), clinical validation costs of biomarkers (especially for dynamic monitoring biomarkers), data privacy and ethical risks of digital health technologies, and the tendency of omics research to “focus on discovery and neglect validation”. Moreover, the common mechanisms (such as universal activation of the interferon pathway) and heterogeneous characteristics (such as T-cell exhaustion subtypes) of diseases revealed by multi-omics studies urgently need to be transformed into universal diagnostic frameworks and stratified treatment strategies. Breaking through these bottlenecks requires establishing interdisciplinary collaboration platforms, developing standardised processes for data sharing and algorithm validation, and promoting regulatory science innovation to balance technological innovation with clinical safety.
